# Fluoroless and contrast-free catheter ablation without a lead apron in routine clinical practice

**DOI:** 10.1038/s41598-020-74165-y

**Published:** 2020-10-13

**Authors:** Takumi J. Matsubara, Katsuhito Fujiu, Yu Shimizu, Tsukasa Oshima, Jun Matsuda, Hiroshi Matsunaga, Gaku Oguri, Eriko Hasumi, Toshiya Kojima, Issei Komuro

**Affiliations:** 1 Department of Cardiovascular Medicine, The University of Tokyo, 7-3-1, Hongo, Bunkyo, Tokyo, 113-8655 Japan; 2 Department of Cardiovascular Medicine, IMS Katsushika Heart Center, 3-30-1, Horikiri, Katsushika, Tokyo, 123-0006 Japan; 3 Department of Advanced Cardiology, The University of Tokyo, Tokyo, Japan

**Keywords:** Cardiology, Interventional cardiology

## Abstract

The technique of catheter ablation has been improved within the past few decades, especially by three-dimensional (3D) mapping system. 3D mapping system has reduced radiation exposure but ablation procedures still require fluoroscopy. Our previous study showed the safety and efficacy of catheter ablation based on intracardiac echogram combined with CARTOSOUND/CARTO3 system, however fluoroscopy use for an average of 16 min is required for this procedure. The present study was aimed to reduce radiation exposure to zero and establish a radiation free catheter ablation method with the goal of utilizing it in routine clinical practice. We conducted single center, retrospective study during 2019 April to 2020 February. Consecutive 76 patients were enrolled. In the first 18 cases, the previously reported procedure (CARTOSOUND/CARTO3 method) was used. The remaining 58 cases were transitioned to fluoroless catheter ablation. The procedure time, success rates and complication rates were analyzed. Not only AF patients but atrial flutter (AFL), paroxysmal supraventricular tachycardia (PSVT) and ventricular arrhythmia patients were included. Catheter positioning, catheter visualization and collecting the geometry of each camber of the heart were conducted by using contact force and ICE based geometry on CARTO system without either prior computed tomography (CT) or magnetic resonance image (MRI). In fluoroless group, all catheter ablations were successfully performed without lead aprons. No complications occurred in either group. There were no significant differences in procedure time in any type of procedure (Total procedure time Fluoro-group; 149 ± 51 min vs. Fluoroless-group; 162 ± 43 min, N.S.), (PSVT 170 ± 53 min vs. 162 ± 29 min, N.S.), (AFL 110 ± 70 min vs. 123 ± 43 min, N.S.), (AF 162 ± 43 min vs. 163 ± 32 min, N.S.). The total radiation time was reduced to zero in fluoroless group. Catheter ablation with ICE and 3D mapping system guide without fluoroscopy could be safely performed with a high success rate, without any prior CT/MRI 3D images. Radiation was reduced completely for patients and staff, negating the need for protective wear for operators.

## Introduction

The technique of catheter ablation has been improved within the past few decades, especially by three-dimensional (3D) mapping system^1^. The details of the anatomy of each chamber of the heart can be reconstructed on the mapping system which assists operators to understand the anatomy of the heart, catheter position and the target site of treatment. The images obtained by enhanced computed tomography (CT) or magnetic resonance image (MRI) is also used for reconstructing the heart 3D images. However, radiation and/or contrast medium are used, in invasive procedures for patients receiving catheter ablation to obtain the detailed information about the heart.

Radiation dose of a single cardiac CT scan is as much as 15 mSv which is almost 7 times higher than the limit for annual natural radiation exposure^2^. Patients who undergo CT scan to reconstruct 3D heart images are exposed to high doses of radiation before receiving catheter ablation. During ablation procedures, additional radiation exposure occurs. In addition to radiation, use of contrast medium is required to perform cardiac CT scan. Contrast medium which used in CT or MRI has deleterious effects on renal function. Its use should be avoided for patients with kidney disease^3,4^. Therefore, there are limitations on using enhanced cardiac CT scan on some group of patients in addition to radiation exposure. MRI also has limitations on patients with non-MRI compatible cardiac implantable devices or claustrophobic patients.

At this point, as reported in our previous study^5^, our previous ablation technique, intra-cardiac echogram (ICE) guided mapping combined with CARTOSOUND/CARTO3 (Biosense Webster, Irvine, CA) system, had already resolved preprocedural CT/MRI associated problems. Our previous procedure does not need prior CT images or MRI images^5^. Moreover, our previous technique also can obtain real-time anatomical information during the procedure. The ICE based geometry technique could avoid the anatomical gap may occurred in merging CT scan images and the images obtained by contact mappings with ablation catheter or mapping catheter^6,7^. Anatomical alteration may occurred due to the lag between CT or MRI scanning and the actual procedure^8^. Regarding contact mappings, catheter contacts resulted in atrial stretching which causes image integration error as previously reported^8^.

Although our previous technique eliminated the need for contrast media and reduced radiation time. However an average of 16 min of radiation exposure to check the position of guide wire or catheter manipulation during the procedure is still required^5^. The exposure of medical staff to radiation remains a risk^9^. Cardiologists have a higher prevalence of malignancies and other non-malignant conditions, and the importance of reducing radiation exposure in both patients and medical staff was mentioned in the ACC/AHA statement^10,11^.

The present study was aimed to completely remove radiation exposure, and establish a radiation and contrast medium-free catheter ablation method as routine clinical practice. There are several reports about fluoroless catheter ablation but in most reports, preoperative cardiac CT scan or MRIs were performed, in tandem with minimal use of fluoroscopy^12–14^. Razminia et al. reported zero-fluoroscopic catheter ablation by using ICE and Ensite system and his procedure has demonstrated the safety of ICE guided catheter ablation^15,16^. The technique reported from Razminia et al. required expert procedure skills and advanced training is necessary. The purpose of this study is to establish the procedure of catheter ablation without radiation and contrast media by using CARTO SOUND system, and to achieve more simplified fluoroless and contrast-free techniques, which can be easily used by many ablation operators.

## Method

### Study population and protocol

The present study was a single center, retrospective study. The observation period was from April 2019 to February 2020. Catheter ablations for 76 consecutive cases were performed by one trained operator. The first 18 cases were scheduled to undergo fluoro-group, our conventional method with fluoroscopy and CARTOSOUND system without prior CT/MRI images^5^. The remaining 58 cases were switched to fluoroless catheter ablation group without prior CT/MRI images, radiation exposure or contrast medium. Protective equipment was also not required for staff.

The procedure time, success rates and complication rates were analyzed. Not only AF patients, but atrial flutter (AFL), atrial tachycardia (AT), paroxysmal supraventricular tachycardia (PSVT) and premature ventricular beats (PVC) patients were also included (Table 1).

**Table 1:** Cases characters.

	Conventional fluoro group (*n* = 18)	Fluoroless group (*n* = 58)
Age	63 ± 11	64 ± 15
Male	15 (83.3%)	38 (65.5%)
PAF	10 (55.6%)	18 (31.0%)
PeAF	1 (5.6%)	14 (24.1%)
LSAF	0 (0.0%)	3 (5.2%)
AFL	5 (27.8%)	13 (22.4%)
AVNRT	2 (11.1%)	6 (10.3%)
AVRT	0 (0.0%)	5 (8.6%)
PVC	0 (0.0%)	2 (3.4%)

*PAF* paroxysmal atrial fibrillation, *PeAF* persistent atrial fibrillation, *LSAF* long standing atrial fibrillation, *AFL* atrial flutter, *AVNRT* atrio-ventricular nodal reentrant tachycardia, *AVRT* atrio-ventricular reentrant tachycardia, *PVC* premature ventricular contraction.

### Procedural description

Preoperative CT and MRI for estimating cardiac geometry were not performed on any of these patients. Trans-esophageal echography was conducted on all AF patients who were above CHADS_2_ score 1 prior to the ablation to check for intra-cardiac thrombus, as any such patients would be excluded from the study. Our patient group contained no one with intra-cardiac thrombus, no patients were excluded from the study.

Esophaster (Japan Lifeline, Tokyo, Japan) was placed in all AF ablation cases for the purpose of thermal monitoring. The position of Esophaster was determined by esophageal electrocardiogram data obtained from the device. Esophaster was visualized on CARTO map during ablation on the posterior wall of the left atrium.

Vascular approach was via the right internal jugular vein and right femoral vein. Punctures were guided by echography. The positions of guide wires were also checked by echogram to confirm the all wires were in the femoral vein. Right femoral artery access was also conducted by the same procedure. 9-Fr long sheath (Abbott, Abbott Park Road, Ill.) was inserted from the right femoral vein, then SOUNDSTAR (Biosense Webster) was inserted into it. During advancement of SOUNDSTAR to the heart, if the target direction was echo free space, ICE was advanced till it reached the right atrium. At the right atrium, ICE was rotated to check whether the other guide wires were situated in appropriate places. When the guide wires were in the inferior vena cava (IVC) or superior vena cava, the rest of the sheaths were inserted. Double 8.5-Fr-SL-0 sheaths (Abbott) for atrial fibrillation (AF), and 8.5-Fr-SL-0 sheath (Abbott) and 8.5-Fr Agillis (Abbott) for AFL were used. 10-Fr of Trio-sheath (Abbott) and 8.5-Fr Agillis (Abbott) were used for PSVT ablation. Guiding sheaths such as SL-0 and Agillis were advanced into the heart by visualized ThermoCool SMART TOUCH ST/SF (Biosense Webster) on CARTO3 system. Anterior–posterior and inferior views were used to check catheter direction, and to deliver ablation catheters into the heart. The presence of the ablation catheter in the IVC was confirmed by checking the axis of the ablation catheter and SOUNDSTAR. If the axis of the ablation catheter was equal to previously delivered ICE, we confirmed that the ablation catheter was in the IVC. Once the ablation catheter had reached the right atrium, contact force sensor was activated. The long sheath is advanced till contact force turns to “SH” which means catheter was stored into the tip of the sheath. Contact force guided sheath delivery was applied to all guiding sheaths. The sheaths positions could be determined without fluoroscopy with this method. BeeAT (Japan Lifeline, Tokyo, Japan), or DecaNav (Biosense Webster) was placed in the coronary sinus (CS), Lasso (Biosense Webster) or Pentaray (Biosense Webster) in the pulmonary vein, SVC, atrium, right ventricular outflow tract and Halo catheter (Johnson & Johnson, New Brunswick, NJ.) was placed around the tricuspid valve. Brockenbrough procedure was performed by radiofrequency needle (RF needle, Japan Lifeline, Tokyo, Japan).

### Intra-cardiac ultrasonography images and intra-cardiac geometry

8 Fr SOUNDSTAR catheter (Biosense Webster) was used to obtain the geometry of each chamber of the heart. All geometrics were generated by the images obtained with ICE. The geometry of each chamber of the heart was reconstructed by collecting the 2D ICE images of inner contours as previously reported^5,8^. For pulmonary vein isolation (PVI) or ablation of the left sided accessary pathway, ICE was introduced into the left atrium after Brockenbrough method was performed. The annular of tricuspid valve or mitral valve was tagged and hollowed out to visualize the edge of the annular during ablation of the cavo-tricuspid isthmus (CTI) or left sided accessory pathway. After geometry generation, ICE was placed in the right atrium to monitor pericardial effusion during the procedure.

### Catheter ablation

Radiofrequency pulses were delivered around each pulmonary vein point-by-point. During AF ablation, output of radiofrequency generator was 50 W and energy delivery time was 7 s for each point^17,18^. Extensive encircling PVI (EEPVI) was performed as standard procedure for PVI. If residual conduction at the carina was detected, carina line was added. End point of AF ablation was to achieve bidirectional conduction block between left atrium and pulmonary vein. If AF persisted after PVI, cardioversion was performed to achieve sinus recovery.

For common AFL, CTI was ablated with 40 W and end point was bidirectional CTI block confirmed by differential pacing maneuver. For PSVT, slow pathway and accessory pathway were ablated with 20 to 30 W each endpoint was a standard endpoint. Heparin was administered by bolus injection and continuous intravenous administration to maintain activated coagulation time 300 to 350 s during procedures.

### Data analysis

Patients backgrounds and history was collected by one author (TJM). The procedure time, success rate and complication rate were analyzed by two-tailed Student’s t test. Values of *p* < 0.05 were considered as significant. Data shown as mean ± SD for normally distributed data. All statistical analyses were conducted by GraphPad Prism version 8 for Mac (GraphPad Software, San Diego, CA).

### Ethics

All methods were carried out in accordance with relevant guidelines and regulations. This study was approved by the institutional ethical committee of the IMS Katsushika Heart Center, and written informed consent was obtained from all participants in this study. Subjects are under 18 was not included in this study.

## Results

### Study population

There were 15 males (83.3%) in conventional fluoro group and 38 males (65.5%) in fluoroless group. Mean age was 63 ± 11 and 64 ± 15, respectively. Other characteristics of the cases were shown in Table 1.

### Catheter positioning and ablation

In 58 cases with fluoroless group, 6 patients required fluoroscopy to deliver guiding sheath into the left atrium or check the guide wire position. Therefore 47 cases were completely zero fluoroscopic ablation.

In all 58 cases with fluoroless group, ICE based geometry was successfully collected.

**Figure 1:**
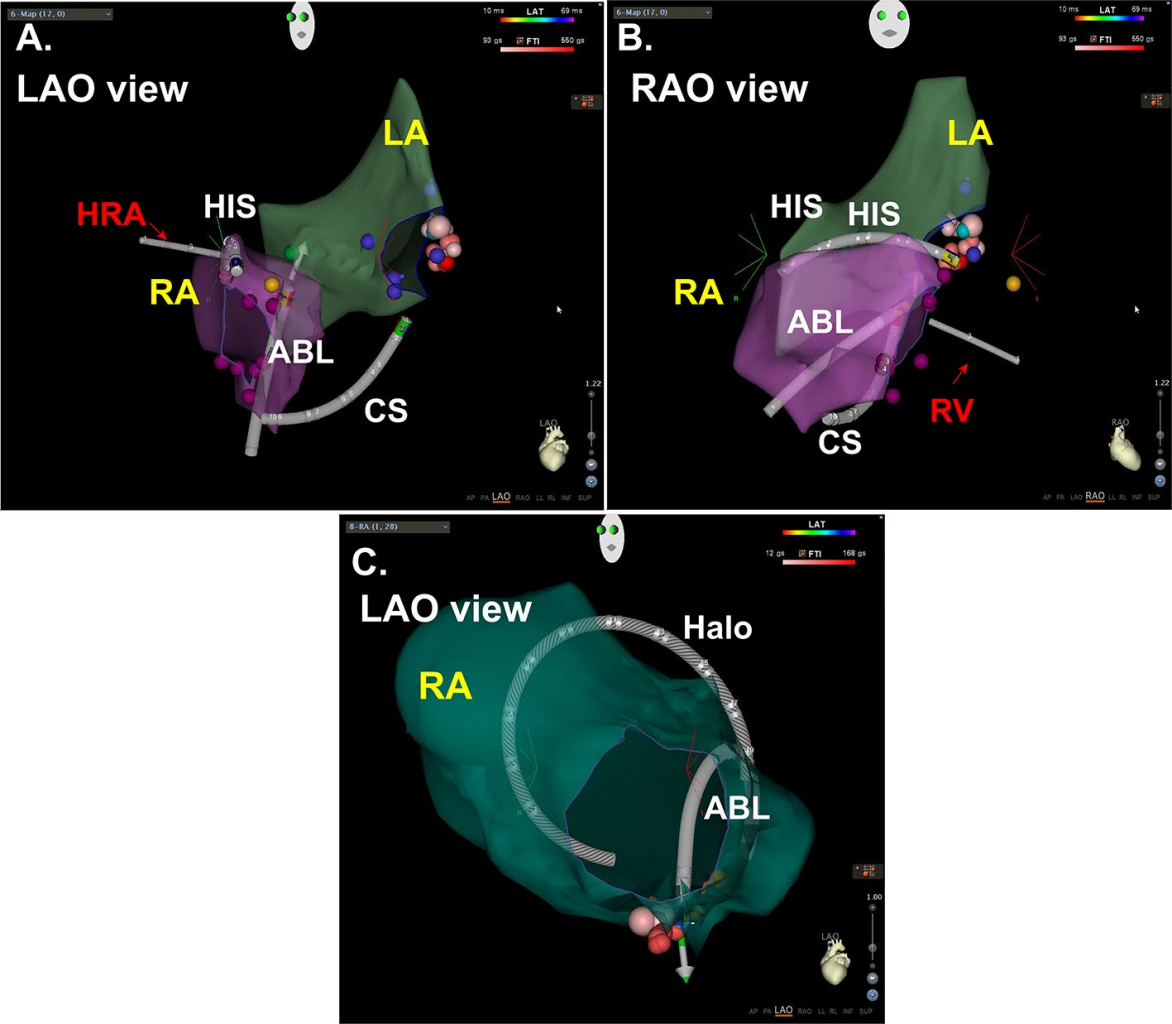
Catheters visualization in case with PSVT and AFL. (**A**) For PSVT case, CS and His catheter was DecaNav. HRA and RV catheter were quatropolar catheter. As HRA and RV catheters could not visualized simultaneously, the cable was switched sequentially to visualize the catheters. (**B**) RV catheter was visualized in RAO view. (**A**, **B**) were same PSVT patient associated with left sided accessory pathway. RA and LA geometry were collected by ICE. (**C**) Halo catheter and ablation catheter were visualized in AFL case. Halo catheter was positioned around tricuspid valve. RA geometry was collected by ICE. RA right atrium, *LA* left atrium, *HRA* high right atrium, *RV* right ventricle, *CS* coronary sinus, *ABL* ablation catheter.

Catheter positioning was performed after placing the ThermoCool ST/SF into the heart to visualize other catheters. CS catheter was delivered in guidance with the CS geometry generated by ICE. For AFL and PSVT, DecaNav (Biosense Webster) was adopted as CS catheter. Halo catheter was placed after ThermoCool ST/SF was delivered into RA. The tetrapolar catheters for EPS were displayed sequentially by switching the cables which are connected to the visualizing port of CARTO system, because catheter display has a limitation to 4 catheters at once. The tetrapolar catheter was positioned at high right atrium (HRA) and right ventricular (RV) during EPS of PSVT. Each catheter positions were successfully displayed on CARTO3 without fluoroscopy in all cases (Fig. 1).

**Figure 2:**
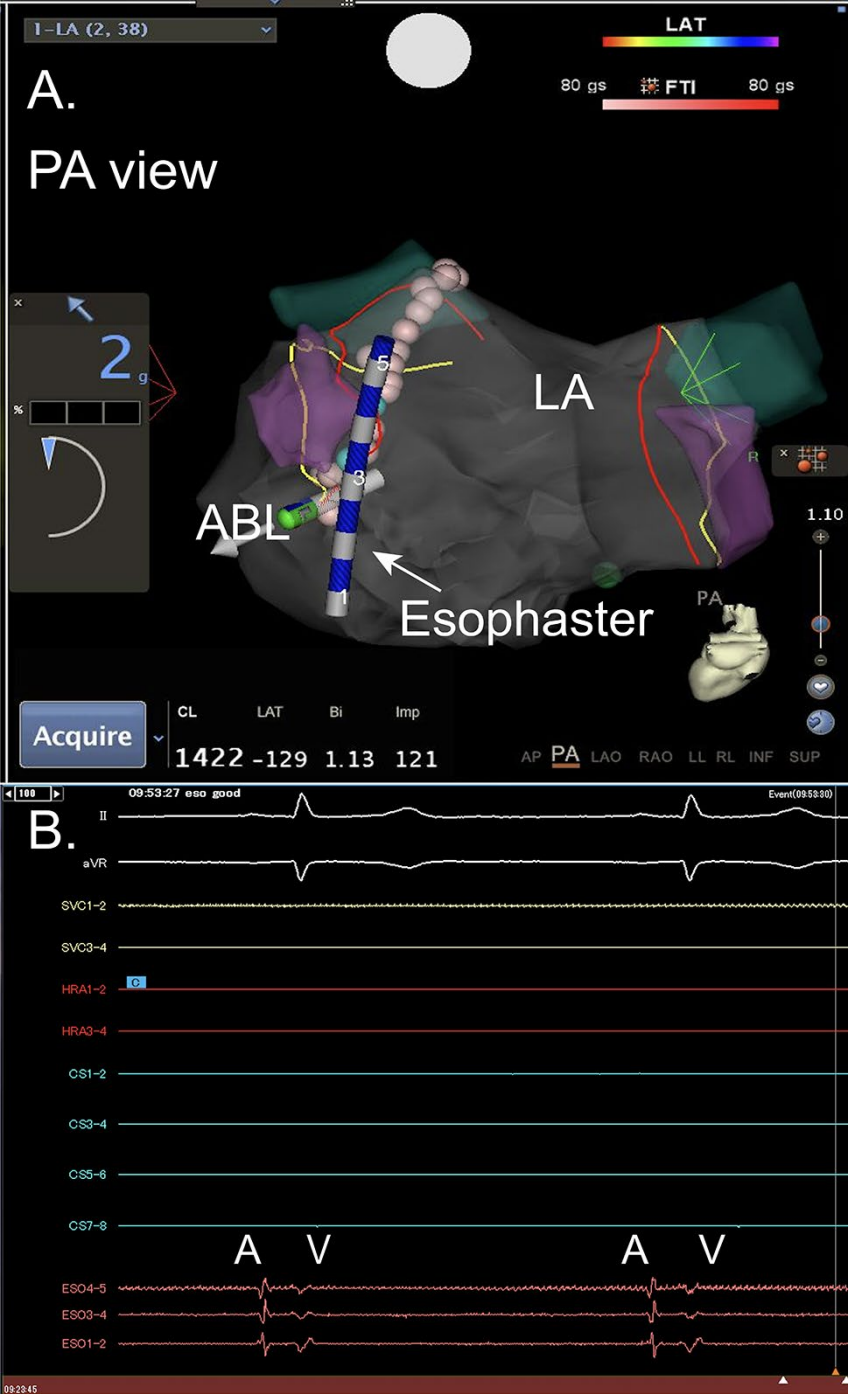
Positioning of Esophaster without fluoroscopy. Esophageal ECG was monitored simultaneously during delivery and precise position was assessed. (**A**) The precise position of Esophaster was considered as the point which (**A**) wave was detected by proximal electrode. During ablation, Esophaster was visualized and if the position was not appropriate, position was adjusted during ablation. (**B**) Electrocardiogram recorded by Esophaster. ESO4-5 was proximal electrode. Esophaster was placed in ideal position when proximal electrode was the fastest site of atrial EGM deflection. (**A**) shows atrial EGM and V shows ventricular EGM. *AP* antero-posterior, *ABL* ablation catheter, *LA* left atrium, *LSPV* left superior pulmonary vein.

At the time of PVI, Esophaster was placed in esophagus by esophageal ECG guide. The position of Esophaster was adjusted during PVI by CARTO image guide (Fig. 2).

**Figure 3:**
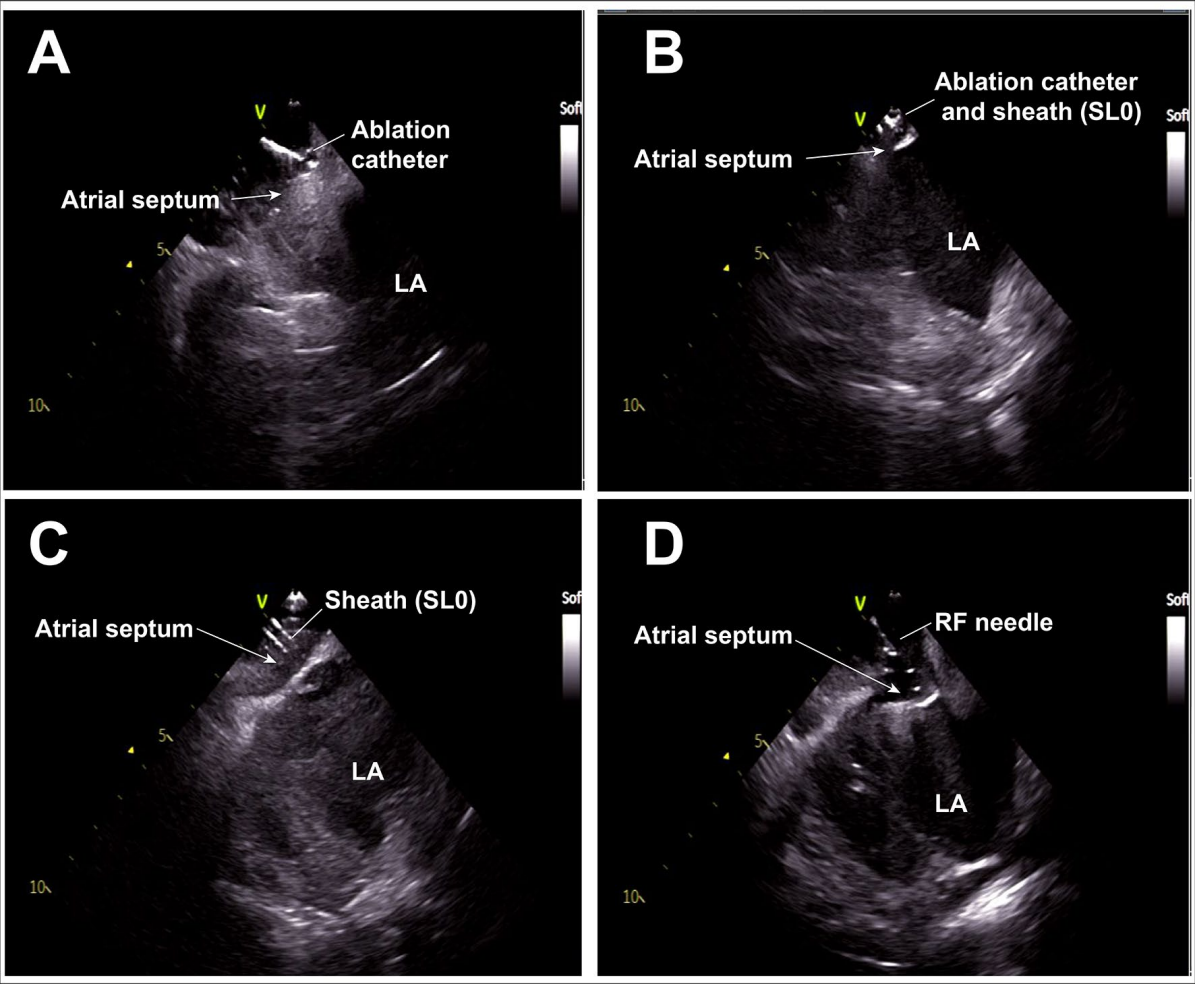
Trans-septal puncture without fluoroscopy. (**A**) The ablation catheter was placed on the atrial septum. (**B**) The sheath (SL0) was advanced till contact force indicator turn to “SH”. (**C**) Ablation catheter was removed from the sheath. Tenting of atrial septum was observed. (**D**) RF needle inserted into inner sheath of SL0 was inserted into the sheath (outer sheath of SL0) and advanced to atrial septum. Tenting of the fossa ovale by RF needle was observed. *LA* left atrium.

Regard to trans-septal approach, SL0 for trans-septal approach was delivered behind the foramen ovale by ThermoCool ST/SF guide. Then ThermoCool ST/SF was exchanged to RF needle inserted into the inner sheath of SL0 precisely to the tip of it. Trans-septal puncture was performed by ICE guide approach; Brockenbrough trans-septal procedure was performed. SL0 was used as guiding sheath during trans-septal puncture. Delivery of SL0 was by ThermoCool guide. As previously mentioned in “Methods”, contact force was monitored during sheath delivery and once ThermoCool was delivered to atrial septum, SL0 was advanced till contact force turned to “SH” which means ThermoCool was situated in the sheath. At this point, the position of SL0 was confirmed by ICE. Then ThermoCool was exchanged with RF needle and trans-septal puncture was performed by ICE guide approach (Fig. 3).

During PVI, Lasso catheter was inserted into the left superior pulmonary vein (LSPV) and ablation as started from the posterior wall of the LSPV antrum. While proceeding the ablation catheter from superior portion of posterior wall to the left inferior pulmonary vein (LIPV) bottom, Esophaster was displayed on CARTO3 to assess the relationship of the esophagus and the ablation line in the posterior wall of LA (Fig. 2). The position of Esophaster was adjusted if the position was inappropriate, based on the visualized Esophaster and LA position on CARTO system. When the antrum of right pulmonary vein (RPV) was ablated, Lasso catheter was inserted to the superior RPV. Bi-directional block was confirmed by pacing from inside of each pulmonary vein. With cases with additional ablation such as BOX isolation of posterior wall, block line was confirmed by voltage and activation mapping by Pentaray catheter. PVI was successfully achieved in all cases.

**Figure 4:**
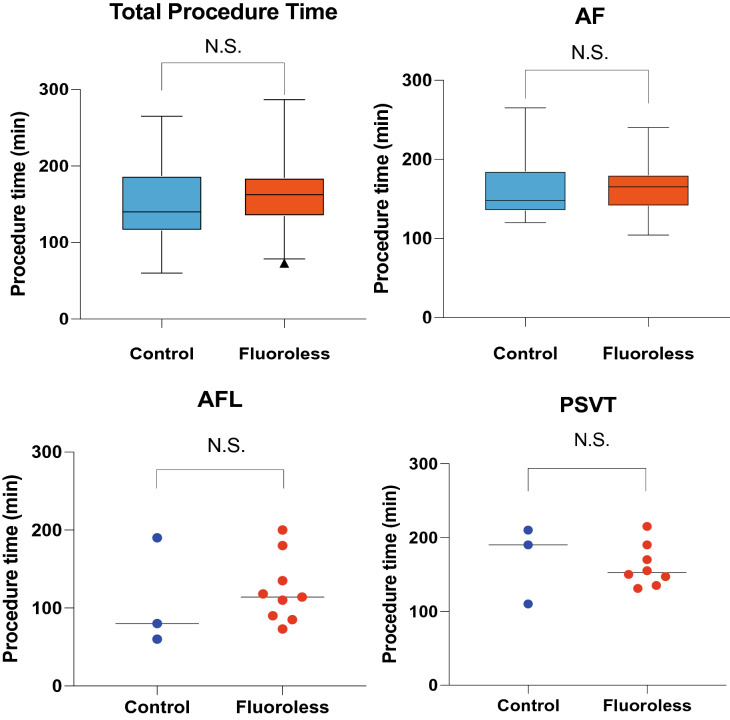
Comparison of procedure time required in conventional fluoro group and fluoroless group. Total procedure time were 149 ± 51 min and 162 ± 43 min in fluoro group vs. fluoroless group. Procedure time in PSVT were 170 ± 53 min vs. 162 ± 29 min. Procedure time in AFL were 110 ± 70 min vs. 123 ± 43 min. Procedure time in AF were 162 ± 43 min vs. 163 ± 32 min. There were no significant differences in fluoro group and fluoroless group. *PSVT* paroxysmal supraventricular tachycardia, *AFL* atrial flutter, *AF* atrial fibrillation, *N.S.* no significant differences.

In all cases, catheter ablation was successfully performed. Procedure-related complications were 0% (Table 2). There were no significant differences in the procedure times between two groups even in any of the procedures (total procedure time fluoro group; 149 ± 51 min vs. fluoroless162 ± 43 min, N.S.), (PSVT 170 ± 53 min vs. 162 ± 29 min, N.S.), (AFL 110 ± 70 min vs. 123 ± 43 min, N.S.), (AF 162 ± 43 min vs. 163 ± 32 min, N.S.) (Fig. 4). The cases with PVC was also successfully performed by fluoroless method. There were no PVC cases in conventional fluoro group. Radiation exposure was completely eliminated in 47 patients in the fluoroless group (81.0%). Average radiation amounts were significantly reduced in the fluoroless group (conventional fluoro group vs. fluoroless group; 149. 5 ± 124.2 mGy vs. 2.9 ± 9.9 mGy, *p* < 0.0001).

**Table 2:** Complications in zero fluoroscopy case.

	Fluoroless group (*n* = 58)
Death	0 (0%)
Cerebrovascular event	0 (0%)
Cardiac tamponade	0 (0%)
Pulmonary vein stenosis	0 (0%)
Hematoma requiring additional procedure	0 (0%)
Ventricular tachycardia	0 (0%)
DVT/pulmonary embolism	0 (0%)
Total	0 (0%)

*DVT* deep vein thrombosis.

## Discussion

Our catheter ablation technique does not require CT or MRI prior to procedure, and nor does it require contrast medium or fluoroscopy. Our procedure also showed a high success rate without complications and no prolongation in procedure time. The low complication rate with fluoroless technique may be due to visualizing the guide wires and the catheters while advancing into the heart to avoid cardiac injury.

In our method, we used the intracardiac echography to evaluate cardiac, vascular anatomy, and procedures with catheters sheathe and wires. However, one thing we did not monitor was the observation of ablated sites. Regard to lesion formation, we adopted high-power short duration (HPSD) radio-frequency ablation. Lesion formation induced by HPSD ablation has been reported, and short duration application of radiofrequency is regarded as a safe procedure from the perspective of avoiding complications such as perforation or thrombus formation at the application sites^17,18^. We believe well-trained operators or upcoming methods would be able to evaluate ablation sites^19–24^.

It was previously reported that it is possible to perform catheter maneuvers with 3D mapping system alone during ablating the heart but before the catheter delivery; fluoroscopy has been used for advancing the guide wires or sheaths to the present time. In the fluoroscopy-guided wire and catheter maneuvers, direction of guide wires or catheters could be assessed but to check positions of the guide wire or the catheter requires multidirectional fluoroscopic view which increases radiation dosage. On the other hand, ICE could clearly view the relationship between the heart chamber and positions of the guide wires or the catheters without fluoroscopy. In addition to ICE view, position of the sheaths could be determined by contact force guide. This study demonstrates that the combination of ICE and contact force maneuver could reduce fluoroscopy.

As several reports regarding reduction of fluoroscopy ablation addressed the major reason of fluoroscopy use was checking the guide wire or catheter position, our method was focused on reducing fluoroscopy during guide wire or sheath delivery and this is one of the key points to achieve fluoroless ablation^13^. Giaccardi et al. reported that their procedure could be used by untrained operator with Ensite system (Abbott) but the procedure requires contact mapping during ablation^13^. As we reported in a previous study, contact mapping could provoke image gaps between 3D anatomy and real anatomy during procedure and it has a risk of cardiac injury. Our method did not require contact mapping and our procedure was non-contact mapping based on ICE. In regard to a risk of cardiac injury, our method could be learned more safely even by untrained operators.

Previous reports regarding the ICE maneuvers for fluoroless ablation requires expert techniques which requires long term training^15,16^. On the other hand, our method required simple techniques which could be applied to routine procedure without special trainings. It is likely that simple techniques will allow ablation operators to learn fluoroless catheter ablation more easily.

We previously reported CARTOSOUND/CARTO3 method^5^. The previous study compared the CARTOSOUND/CARTO3 method to the conventional procedure performed with CARTO Merge system. The safety and efficacy of the CARTOSOUND/CARTO3 method were ascertained. Radiation or use of contrast media was reduced due to the elimination of the CT scan before the procedure. In terms of the required amount of contrast media, we did not need contrast through the entire procedure. However, radiation was still required for the CARTOSOUND/CARTO3 method. The present method can eliminate radiation by further utilization of the image of the SOUNDSTAR and CARTOSOUND 3D mapping system.

The learning curve of the CARTOSOUND/CARTO3 method was reported in the study, and only 8 cases were required for operators to learn the method^5^. We believe the technique could be acquired without difficulty in a short period.

The present new method is based on visualizing the guidewire and the sheath by ICE. The procedure, manipulation of ICE without fluoroscopy, has been reported from Razminia et al., and the report showed the safety of ICE manipulation^15,16^. The ICE technique was simple as we had initially expected; advance the ICE when the direction of interest was echo-free space. With this manipulation, the vascular injury was avoided. Although further study is required to estimate the learning curve in early career operators, we assumed that learning the present method in early career operators also possible.

If the operators performed fluoroless procedure as routine practice, adverse effects of radiation could be reduced both in patients and medical staff.

## Conclusions

Catheter ablation with ICE and 3D mapping system guide, without fluoroscopy and without any prior CT/MRI 3D images, could be safely performed with high success rate. Radiation was reduced completely for patients and staff, negating the need for protective equipment for operators.

## Study limitation

The present study had a small number of patients, safety of fluoroless ablation with CARTO system should be assessed with a large number of patients. Acute success rates were high in the present study but long term results such as recurrence rates should be addressed in the future.
